# Besides and beyond Flowering: Other Roles of *EuAP2* Genes in Plant Development

**DOI:** 10.3390/genes10120994

**Published:** 2019-12-01

**Authors:** Charles U. Solomon, Sinéad Drea

**Affiliations:** 1 Department of Genetics and Genome Biology, University of Leicester, Leicester LE1 7RH, UK; 2 Department of Plant Science and Biotechnology, Abia State University, PMB 2000, Uturu 441107, Nigeria

**Keywords:** *euAP2* genes, flowering, plant development

## Abstract

*EuAP2* genes are well-known for their role in flower development, a legacy of the founding member of this subfamily of transcription factors, whose mutants lacked petals in *Arabidopsis*. However, studies of *euAP2* genes in several species have accumulated evidence highlighting the diverse roles of euAP2 genes in other aspects of plant development. Here, we emphasize other developmental roles of *euAP2* genes in various species and suggest a shift from regarding *euAP2* genes as just flowering genes to consider the global role they may be playing in plant development. We hypothesize that their almost universal expression profile and pleiotropic effects of their mutation suggest their involvement in fundamental plant development processes.

## 1. Introduction

*APETALA2* (*AP2*) genes are named after a series of *Arabidopsis* mutants characterized by homeotic transformations of their sepals to leaves and/or carpels and petals to staminoid petals. Analysis of the *AP2* mutants, along with other floral mutants, gave birth to the ABC model of flower development where AP2 is classified as an A-class gene [1,2].

The forerunner AP2 protein was cloned and characterized in *Arabidopsis* [3]. The *Arabidopsis* AP2 protein comprising 432 amino acids (aa) is mainly characterized by the possession of two AP2 domains, each made up of 68-aa with an 18-aa core conserved section that forms an amphipathic α–helix. The two AP2 domains, called AP2-R1 and AP2-R2 (R for Repeat), have 53% amino acid identity and 69% amino acid homology. Their 18-aa core conserved sections show 83% amino acid homology [3]. Sequence analysis of the *AP2* gene showed that it has a domain that can activate RNA polymerase II transcription factor and another domain that is a putative nuclear localization signal. The presence of these domains served as evidence to suggest that the AP2 protein is a transcription factor [3,4].

**Figure 1:**
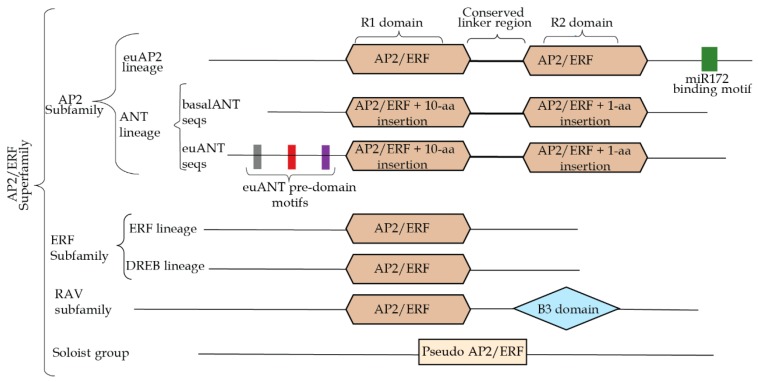
Structure of the *APETALA 2* (AP2)/ethylene response factor (ERF) transcription factor superfamily. EuAP2 genes belong to the AP2 subfamily. They are distinguished from (ANTEGUIMENTA) ANT lineage genes by the presence of miR172 binding site. Not to scale. Adapted from [12,15].

Following the cloning and characterization of the *AP2* gene, other genes encoding two AP2 domains were identified in *Arabidopsis* [5,6,7]. About the same time, ethylene-responsive element binding proteins (EREBP) from tobacco were shown to contain a conserved DNA-binding domain [8]. Sequence comparison by alignment of EREBP, also known as ethylene responsive factor (ERF), and AP2 domains revealed they were related [4,6]. This relationship subsequently led to the classification of genes having AP2/EREBP domains into one superfamily of transcription factors called APETALA 2/ethylene response factor (AP2/ERF) [9,10]. The AP2/ERF superfamily is divided into the following four subfamilies based on the number of AP2 domains and sequence similarity:

- AP2: Genes that belong to this subfamily have two AP2 domains connected by a linker region of about 20-aa. They are further divided into euAP2 and (ANTEGUIMENTA) ANT AP2 lineages [11,12]. The distinction between these lineages is based on 10-aa and 1-aa insertion found, respectively, in the R1 and R2 domains of ANT AP2 genes that are absent in euAP2 genes. An additional distinction is the presence of a miR172 binding site in euAP2 lineage that is absent in ANT AP2 [12]. The ANT AP2 lineage is also further divided into euANT sequences that possess three additional pre-AP2 domain motifs and basal ANT sequences that lack such motifs (Figure 1) [12,13].
- ERF: This subfamily is comprised of genes that have single AP2 domain. It is usually the largest subfamily within the ERF/AP2 superfamily in most plant species whose genomes have been studied. It is subdivided into ten groups, broadly divided into dehydration-responsive element binding-proteins (DREB) comprising groups I–IV and ethylene response factor made up of groups V–X [9,10,14].
- RAV (RAV for related to AB13/VP1): This subfamily is characterized by the possession of a B3 domain in addition to a single AP2 domain [9,14,15].
- Soloist: This subfamily is comprised of genes with domain sequences that closely resemble the AP2 domain but are too diverged and lack other features that can qualify them to be classified into any of the other subfamilies [11].

Genes with AP2/ERF domains were initially thought to be plant specific. However, genes with similar domains have been confirmed to exist in ciliates, bacteriophages and cyanobacteria [12,16,17]. Genes belonging to each subfamily, except soloist, have been shown to recognize and bind to different DNA sequences. AP2 binds to 5′-GCAC(A/G)N(A/T)TCCC(A/G)ANG(C/T)-3′, DREB binds to 5′-A/GCCGAC-3′, ERF binds to 5′-AGCCGCC-3′, and the AP2/ERF domain of RAV binds to 5′-CAACA-3′. The conserved linker region between the two domains of the AP2 subfamily is critical for DNA binding [18,19,20]. Functional analysis of proteins belonging to the AP2/ERF superfamily suggests that while genes belonging to the AP2 and RAV subfamilies are generally involved with developmental processes, ERF subfamily genes have been largely implicated in stress response processes [12,19].

The AP2/ERF superfamily of transcription factors is one of the largest in most plant species whose genome sequences have been analyzed [21]. Starting with *Arabidopsis*, genome-wide analysis of AP2/ERF genes have been performed for a number of plant species. Some of them are presented in Table 1. However, the scale, scope and aim of most of the studies that describe AP2/ERF transcription factors in genomes of various plant species often neglect detailed clade-specific phylogenetic analysis of each subfamily. Hence the actual number of *euAP2* genes is not yet known in most plant species.

The forerunner *Arabidopsis* AP2 protein belongs to the euAP2 lineage. Genome-wide analysis showed that the euAP2 lineage is made up of six genes in *Arabidopsis* [12]. These six genes have been actively studied in the context of their role in floral ontogeny. They have been linked with aspects of flowering such as flowering time, floral meristem identity and flower morphology [51,52,53]. For recent updates on the ABC floral model see [54,55,56,57] and references therein. However, functional characterization in *Arabidopsis* and several other plants indicate that *euAP2* genes are involved in other developmental processes besides flower development. Here, we present a summary of their expression profiles in various plant species, and attempt to summarize evidence that underscores the roles of *euAP2* genes in other aspects of plant development. By highlighting other roles of *euAP2* genes in plant development, we aim to bring attention to their possible involvement in global and fundamental plant developmental processes.

## 2. Expression of *EuAP2* Genes

**Figure 2:**
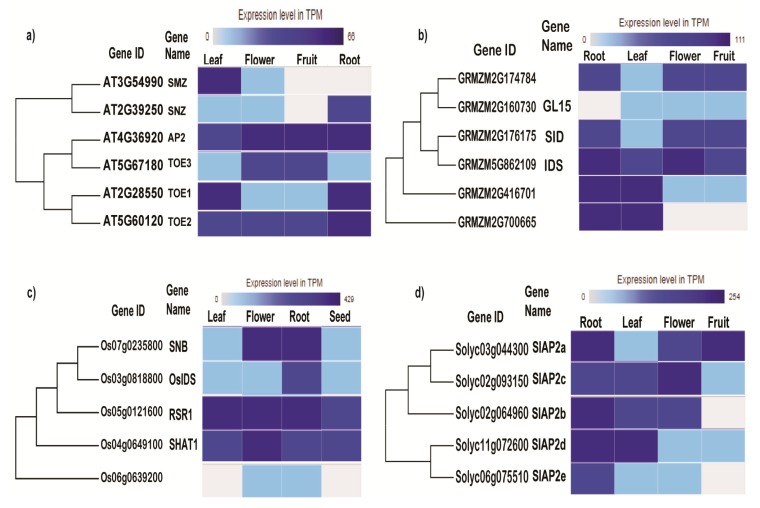
Expression profile of euAP2 genes in selected monocot and dicot species. The expression profile of euAP2 genes in root, leaf, flower and fruit of: (**a**) *Arabidopsis*, (**b**) maize, (**c**) rice, and (**d**) tomato. Irrespective of species, some euAP2 genes are expressed in all the tissues surveyed, while one or two are not expressed in some tissues. The expression profiles were sourced from Expression Atlas from the following experiments: *Arabidopsis* [63], maize [64], rice [65] and tomato [66].

*EuAP2* genes are found expressed in major tissues (Figure 2). However, there are differences in the expression profiles of individual genes. Their expression profiles suggest a prominent gene that is more highly expressed in all tissues compared to others. This gene is called *AtAP2* in *Arabidopsis*, *INDETERMINATE SPIKELET* (*IDS*) in maize, *RICE STARCH REGULATOR 1* (*RSR1*) in rice, *Q* in wheat, and *SlAP2a* in tomato. These genes have been functionally characterized in the species listed. From such studies, we learn that mutations in this prominent euAP2 gene leads to dramatic and ‘easily observed’ phenotypes [3,58,59,60]. Mutations in other *euAP2* genes that are expressed quite broadly but less highly than the prominent *euAP2* gene lead to no or less-pronounced phenotypes. This has prompted the suggestion that they play redundant roles [53,61]. Interestingly, one or two *euAP2* gene(s) in various species are not universally expressed (Figure 2). They may be found not expressed in one or two organs. A loose consensus is that they are not expressed in mature fruits and seeds. However, the mRNA expression profile of *euAP2* genes should be interpreted carefully because miR172 has been proven to regulate the translation of euAP2 mRNA into protein [51,52,61,62].

## 3. Briefly on miR172 Regulation of *EuAP2* Genes

MicroRNAs (miRs) are short endogenous RNA sequences (approximately 22 nt in length) that are involved in post-transcriptional regulation of gene expression. First discovered in *Caenorhabditis elegans*, miRs are now known to be present in all the major plant lineages [67]. *EuAP2* genes are regulated by miR172. The mechanism of miR172 regulation of *euAP2* genes can be either by cleavage of euAP2 mRNA to smaller fragments detectable by PCR, or inhibition of translation of euAP2 mRNA to protein [51,52,68].

A careful study of the literature reporting miR172 regulation of *euAP2* genes suggests a seeming pattern of partial or total tempo/spatial regulation of *euAP2* genes by miR172 at critical steps in the development of plant reproductive tissues. Apparently, *euAP2* genes are freely expressed in various tissues during early vegetative growth phase. However, as a plant approaches reproductive phase, miR172 is recruited to regulate expression of *euAP2* genes in a timely and spatially restricted manner leading to the development of normal reproductive tissues [51,52,61,62,68,69]. Hence, ectopic autologous and heterologous overexpression of miR172 interrupts the vegetative growth phase activities of *euAP2* genes and leads to precocious transition to reproductive phase in plants [53,70]. MiR172 regulation of *euAP2* genes is very efficient even when *euAP2* genes are constitutively overexpressed [52,53]. However, the regulatory ability of miR172 on *euAP2* genes is very sensitive to base mismatches on the complementary binding sequence on euAP2 mRNA. One base substitution on the miR172 binding site is enough to render an *euAP2* gene resistant to miR172 regulation [68]. On the other hand, miR172 is regulated by *euAP2* genes in a negative feedback loop [71]. Remarkably, the regulatory effects of miR172 have been shown to be graft-transmissible in potato, prompting the suggestion that miR172 is either mobile or can regulate *euAP2* genes through long-distance signaling [72]. Whatever the mechanism, this observation warrants similar studies in perennial tree species that are amenable to grafting, because it hints at the possibility of downregulating *euAP2* genes in non-transgenic plant stocks by grafting miR172-overexpressing scions. Furthermore, the discovery that primary transcripts of miRs (pri-miRNAs) also encode for small peptides called miPEPs is exciting and holds lots of potential in the study of *euAP2* genes [73]. These miPEPs positively stimulate the transcription of their corresponding pri-miRNAs thereby increasing the regulatory effects of miRs on target transcription factors. Crucially, it has been demonstrated that exogenous application of synthetic miPEP172c increases the transcription of miR172c which in turn downregulates the *euAP2* gene *NODULE NUMBER CONTROL 1* (*NNC1*), leading to an increase in nodule numbers in soybean [74].

The evidence available so far suggests that miR172 only regulates *euAP2* genes [53]. Therefore, one may be safe to assume that the outcome of experiments where miR172 is constitutively overexpressed will be identical to the outcome of an experiment where all the *euAP2* genes in a plant are knocked out. Indeed, Ref. [53] showed that hexuple-null mutant of *Arabidopsis euAP2* genes phenocopied constitutively overexpressed miR172 in flowering time. Therefore, results obtained by [72,75,76,77,78,79], from experiments where miR172 was constitutively overexpressed, are equivalent to the loss of function of entire *euAP2* genes in the plant species studied.

## 4. Roles of *EuAP2* Genes in Plant Development

### 4.1. EuAP2 Genes are Negative Regulators of Plant Height

From herbs to trees, plant height is considered an important trait. It is a good indicator of access to light, biomass accumulation, and how well a plant is able to resist physical forces like wind. Plant height is usually measured on the vertical axis from soil level to the apex of the main stem. Plant height is therefore subject to the proliferative activity of stem cells in the shoot apical meristem. *AtAP2* is expressed in the shoot apical meristem, and along with *WUSCHEL* (*WUS*) and *CLAVATA3* (*CLV3*) function in stem cell maintenance [80]. The role of *AtAP2* in stem cell maintenance was discovered with *l28*, a dominant-negative allele of *AtAP2* harboring a single nucleotide polymorphism that changed Glu to Lys in the first AP2 DNA-binding domain [81]. This *l28* causes a dosage-dependent premature termination of primary shoot meristem in heterozygous diploid and triploid mutants. Homozygous *l28* mutants produced no rosette leaves and had an astounding 99.7% frequency of shoot meristem termination, resulting in very short plants that died a few days after germination [80,81]. Although there is no quantitative data, image data suggest that *AtAP2* and other *euAP2* genes also regulate plant-height dynamics in *Arabidopsis*. In [53,82], single and multiple null-*euap2* mutants resulted in taller early flowering plants relative to wild type. It will be interesting to know how the final plant height of these mutants compares to that of wild-type plants.

Patil et al. [83] recently reported that plant height is repressed in *Zeo1.b* barley mutants compared to wild type. *Zeo1.b* plants overexpress *HvAP2* because they possess an allele of *HvAP2* with a single nucleotide polymorphism (SNP) in the miR172 binding site, and are, therefore, resistant to miR172 regulation [68]. Patil et al. [83] showed that by seven weeks after planting, the height of *Zeo1.b* plants was only about 50% of the height of wild-type plants. Their analyses indicate that the reduced plant height in *Zeo1.b* may be attributed to fewer internodes and less number of cells in the peduncle compared to wild type.

### 4.2. SlAP2a is a Negative Regulator of Tomato Fruit Ripening

Functional analyses have shown that *Solanum lycopersicum APETALA2a* (*SlAP2a*) regulates aspects of tomato fruit development and ripening in two similar but independent studies [59,84]. In both studies, expression of *SlAP2a* was suppressed using RNA inhibition (RNAi). *SlAP2a-RNAi* fruits ripened about seven days earlier than wild-type fruits, turning an uneven orange/yellow color, while wild-type fruits were uniform red in color when ripe [59,84]. In these two studies, the investigators showed that the observed differences in the pigmentation of ripe tomato fruits could, in addition to other factors, be attributed to an increased −β–carotene to lycopene ratio in *SIAP2a-RNAi* fruits compared to the wild type. Ethylene production was found to be higher in *SIAP2a-RNAi* fruits relative to wild type. Fruit softening and disintegration was also observed to occur earlier and more rapidly in *SIAP2a-RNAi* tomato fruits than in wild-type fruits [84]. Mature green tomato fruits of *AP2i-RNAi* lines had abnormal shape with indentations and an uneven surface that split open when ripe compared to wild-type fruits which were round in shape and had a smooth surface [84]. These observations were recently confirmed in null-*ap2a* mutants generated using clustered regularly interspaced short palindromic repeats (CRISPR)/CRISPR-associated protein 9 (Cas9)-mutagenesis [85].

### 4.3. EuAP2 Genes are Negative Regulators of Seed Size and Affect Seed Quality

In similar studies, conducted around the same time, two groups reported that *AtAP2* influenced seed shape, size, mass, content and yield in *Arabidopsis* [86,87]. Seeds of *ap2* mutant plants were larger in size and had more weight compared to wild-type seeds. Increase in seed weight and size in *ap2* plants were also accompanied by an increase in total seed protein and total seed oils content compared to wild-type seeds. However, fewer seeds were produced in *ap2* siliques relative to wild type [86,87]. Both groups also reported that *ap2* mutant embryos had more, larger and irregularly shaped cells compared to wild-type embryos. They concluded that *AP2* affects embryo cell number and size. In addition, *AtAP2* is also known to play roles in seed coat morphology. [3,88]. The seed epidermal cells of *ap2-6*-null mutants are rectangular in shape, contrasting hexagonal-shaped epidermal cells of wild-type seeds. Developmental analysis by [88] revealed that the outer integument development proceeds normally in *ap2-6* seed coats until about four days after pollination (DAP). At this point, further differentiation is terminated, so that at maturity, epidermal and sub-epidermal cell types and structures such as columella are absent. Consequently, mucilage synthesis, storage and secretion are absent or very limited in the seed coat of *ap2* seeds [3,88,89]. Since *AP2* acts maternally, this altered seed morphology and content may be attributed to altered composition of sugar reaching the developing seeds from the mother plant [87]. Sugar analysis revealed that *ap2* mutant seeds had a higher hexose to sucrose ratio relative to wild type seeds during development. Hexoses fuel metabolic reactions and cell division. Their presence in a higher concentration for a longer time during *ap2* mutant seed development may contribute to the increase in number and size of cells.

Three rice *euAP2* genes, *SHATTERING ABORTION1* (*SHAT1*), *RICE STARCH REGULATOR 1* (*RSR1*) and *SUPERNUMERARY BRACT* (*SNB*), have been reported as negative regulators of rice seed size [90,91,92]. Grains from null or RNAi mutants of *SHAT1*, *RSR1* and *SNB* were longer in length and weighed more relative to wild-type grains. Their overexpression, on the other hand, resulted in shorter grains with lower weights compared to wild type. Although the loss of these genes also resulted in reduced seed-setting rate, overall yield was however improved. The histological basis of increased grain length in *ssh1* was due to increased cell size and not increase in cell number. Similarly, wheat grain length and weight increased in test plants relative to control by barley stripe mosaic virus—virus-induced gene-silencing (BSMV-VIGS) of wheat starch regulator 1 (*TaRSR1*) [93]. Wheat grain morphology is also controlled by *Q*, a major domestication gene [94]. The *Q* allele originated from a single nucleotide polymorphism in the miRNA172-binding site of the wild type *q* allele. No longer subject to miR172 regulation, *Q* is an overexpressed *euAP2* gene [94,95]. Expectedly, the grains of wheat plants possessing *Q* are shorter and rounder compared to plants with *q*. However, *Q* also contributed to higher grain weight and yield, also had lower seed-setting rate compared to *q* [96]. Remarkably, whereas loss-of-function and gain-of-function mutations in rice *euAP2* genes resulted in opposite phenotypes in grain weight and yield, it appears that both gain/loss-of-function mutations of *Q* result in similar grain weight and yield phenotypes. Therefore, the effects of *euAP2* genes on grain filling appear to differ between rice and wheat. Curiously, rice and wheat *RSR1* have been functionally characterized as negative regulators of a subset of starch synthesis related genes that are highly expressed in the endosperm [91,94]. Therefore, it is rather interesting that *Q* does not inhibit starch synthesis in wheat. It will be also interesting to see how the overexpression of *TaRSR1* will affect starch synthesis and grain weight in wheat.

The effects of *Q* in wheat grain processing quality was recently reported by [97]. They mapped a new allele of *Q* called *Qc1* from a wheat mutant (*S-Cp1-1*) characterized by dense spike. Their results demonstrated higher significant values in four wheat grain processing parameters in the mutant compared to wild type. Remarkably, the new allele correlated with about 60 g kg^−1^ increase in grain protein content (GPC) compared to *Q*. When used to make bread, loafs from the *Q* mutant dough were larger compared to wild type [97].

### 4.4. EuAP2 Genes are Negative Regulators of Phase Change

The life cycle of a plant occurs in phases such as dormant seed phase, juvenile vegetative growth phase, adult vegetative growth phase and reproductive phase. While transition from one phase to another may be marked by appearance of tissues that were hitherto absent in the plant, phase change is also often characterized by anatomical, physiological and morphological differences between identical organs already formed in the previous phase and those that develop in the new phase. This phenomenon is known as heteroblasty [98,99]. Following germination, an *Arabidopsis* plant usually produces rosette leaves separated by short internodes. Then, the internode elongates, producing cauline leaves along the way before terminating in inflorescence [100]. The differences between *Arabidopsis* rosette and cauline leaves demonstrates heteroblasty. The timing and sequence of developmental phases in plants is influenced by genetic and environmental factors. Changes in developmental timing is called heterochrony and mutations that alter developmental timing are said to be heterochronic.

*EuAP2* genes have been associated with leaf heteroblasty in *Arabidopsis* and maize [51,52,98,101]. *Arabidopsis* null mutants for *euAP2* genes produce lesser number of rosette leaves compared to wild-type plants. This was observed in single and multiple null *ap2* mutants. However, the number of cauline leaves produced were identical between multiple null *ap2* mutants and wild-type plants. In addition, hexuple-null *ap2* mutant plants showed early formation of trichomes on their lower leaf surface signifying precocious transition from vegetative to reproductive phase [53].

Phase-change related heteroblastic and heterochronic effects of *Glossy15* (*GL15*) on maize leaves is well-documented [98,101]. Post-germination, a maize plant will first produce 5–6 juvenile leaves. Subsequent leaves are called adult leaves. Maize juvenile and adult leaves are distinct in some features such as cell wall characteristics, epidermal cell morphology, fine structure and histo-chemistry of epicuticular waxes. Overexpression of *GL15* leads to an increase in the number of juvenile leaves and a delay in transition from vegetative to reproductive phase [98]. Furthermore, timely regulation of *HvAP2* by miR172 is required for barley rachis elongation [68]. This was revealed in the barley mutant *Zeo1.b*, which has an allele of *HvAP2* that is resistant to miR172 regulation. The dense spike of *Zeo1.b* mutants results from heterochronic variation in the degradation of *HvAP2* by miR172.

**Figure 3:**
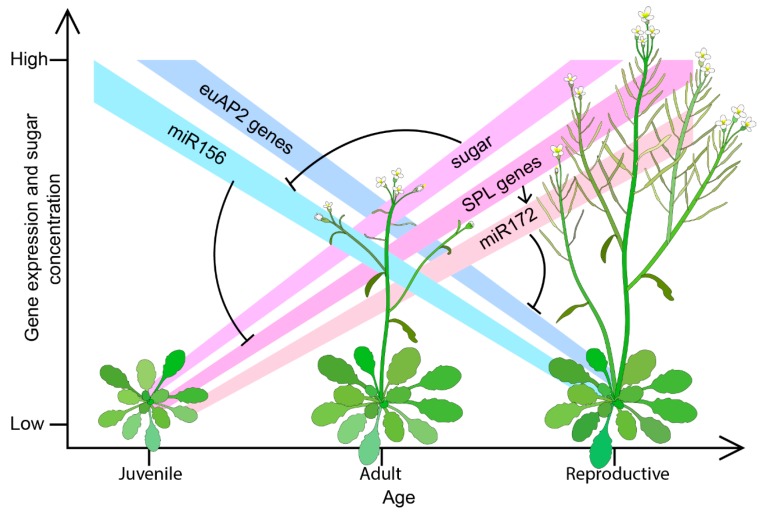
Phase-change regulation in plants. EuAP2 genes are part of the regulatory complex that regulates phase change in plants.

The interactions between *euAP2* genes, miR172, *SQUAMOSA PROMOTER BINDING PROTEIN LIKE* (*SPL*) genes and miR156 is considered crucial in the regulation of vegetative phase change in plants. Just like miR172 targets only *euAP2* genes among *AP2-like* genes, miR156 targets specific members of *SPL* genes. Early in plant development, miR156 is highly expressed leading to the repression of its *SPL* targets. As the plant develops, it accumulates sugars which downregulates miR156 resulting in increased expression of its target *SPL* genes. Among the *SPL* genes regulated by miR156, *SPL9* and *SPL10* in *Arabidopsis* are known to upregulate miR172, which in turn downregulates *euAP2* genes leading to vegetative phase change (Figure 3) [71,102,103,104].

Furthermore, *euAP2* genes also participate in the regulatory complex that decides when a plant should stop flowering and terminate the reproductive phase. Balanzà et al. [105] reported that global proliferative arrest (GPA) is delayed in *Arabidopsis* loss-of-function mutants of *FRUITFULL* (*FUL*), a MADS-box gene and *AP2* gain-of-function mutants. Their analysis showed that *AP2* acts downstream of *FUL* and that *FUL* is able to downregulate *euAP2* genes by binding directly to their promoters. They further showed that *FUL*-mediated transcriptional inhibition of *euAP2* genes in the shoot apical meristem results in the downregulation of *WUS* and thereby the loss of stem cell maintenance that precipitates plant death in monocarpic plants.

### 4.5. EuAP2 Genes are Positive Regulators of Shattering

Shattering, also referred to as dehiscence, is a dispersal mechanism employed by some plants whose fruits are dry at maturity. To achieve the dehiscence of an organ, a specialized abscission zone (AZ) (also known as dehiscion zone (DZ)) usually differentiates between the organ and the mother plant. Cells that make up the AZ start out small and dense compared to neighboring cells. At the time of abscission, however, they have enlarged and accumulated lignin. In *Arabidopsis*, a separation layer is sandwiched between the lignified replum and lignified valve margin [106]. A cocktail of cell wall remodeling enzymes such as polygalacturonase, cellulase and xyloglucan endotransglycosylase anchor on the lignified cell walls and dissolve the middle lamella to accomplish shattering. Furthermore, drying-induced mechanical tension between the lignified cells may contribute to shattering [107,108,109].

In *Arabidopsis* whose fruit is a silique that disperses its seeds by dehiscence, replum and valve margin cells were larger and more lignified than in null *AtAP2* mutant fruits compared to wild type. Consequently, there was slight delay in dehiscence of *AtAP2* fruits [106]. Two *euAP2* genes in rice, *SHATTERING ABORTION1* (*SHAT1*) and *SUPERNUMERARY BRACT* (*SNB*), have been characterized as positive regulators of shattering [91,92]. Both genes affect the differentiation of the AZ. The AZ does not differentiate in *shat1* mutants. Although the AZ differentiates in null *SNB* mutants, *suppression of shattering1* (*ssh1*) and in *RNAi-SNB*, lignin deposition was higher in these mutants compared to wild type. Additionally, lignin biosynthesis genes were differentially expressed in young *ssh1* panicles. Interestingly, lignin deposition also appears to be higher in *OE-SNB* AZ compared to wild type [91]. Overexpression of *HvAP2* also results in over deposition of lignin in barley peduncle [83]. Therefore *euAP2* genes may be important regulators of lignin synthesis and deposition throughout the plant.

One of the many functions of wheat *Q* gene is the conferment of the non-shattering trait on modern wheat cultivars [60]. Recently, a new *Q* allele (Q^t^), distributed only in Tibetan semi-wild wheat populations, with an 161-bp transposon insertion in exon 5 was characterized [110]. While the expression of Q^t^ was comparable to wild-type *Q*, Q^t^ protein function was impaired, resulting in shattering. Therefore, the insertional mutation led to loss-of-function of *Q*, and results in de-domestication. Histological analysis revealed that non-shattering wheat lines had less lignin deposit on rachis cells than shattering lines. It will be interesting to investigate the molecular differences between *q*-mediated shattering and non-shattering *Q*.

### 4.6. EuAP2 Genes are Negative Regulators of Cleistogamy in Grasses

Flowers that are self-pollinated because they remain closed at maturity are cleistogamous. In cereals like barley, wheat and rice, a pair of lodicules below the carpel swell just before anthesis, forcing the lemma and ovary apart, which results in open (chasmogamous) flowers at anthesis. Two lobes, a lower extensible cushion lobe and a thin feathery upper lobe, make up the lodicule [111,112]. The lodicule has extensive vascularization through which assimilate (mostly sugar) is rapidly imported to affect lodicule enlargement at anthesis.

*Cleistogamy1* (*Cly1*) (also known as *HvAP2*) encodes a miR172-resistant *euAP2* gene and is therefore overexpressed. The lodicule differentiates fully in *Cly1* plants but does not enlarge enough at anthesis to open the floret and is thus cleistogamous [113,114]. While current evidence suggests that sugar importation into the lodicule is restricted in *Cly1* plants, there is need for further histological and physiological investigation of sugar importation into *Cly1* lodicules. Conversely, *shat1* loss-of-function mutants had larger and sometimes more lodicules compared to wild-type rice [92]. In addition, overexpression of miR172 which effectively inhibit *euAP2* genes also led to increased lodicule size and number in rice [76]. Thus, the role *euAP2* genes in lodicule expansion may be conserved across grass species.

### 4.7. EuAP2 Genes are Negative Regulators of Nodulation and Tuberization

The regulatory activities of *euAP2* genes have also been observed underground where they function as negative regulators of nodulation and tuberization. Legumes are able to utilize atmospheric nitrogen by accommodating nitrogen-fixing bacteria in specialized root structures called nodules. The formation and maintenance of nodules is an energy demanding, highly regulated process involving communication between the colonizing bacteria and the host legume. Prior to nodule initiation, several genes are upregulated in response to lipochito-oligosaccharide signals (known as nodulation factors (NFs)) released by the rhizobia [115,116]. Among the proteins upregulated downstream of the NFs response cascade include small, mobile CLAVATA/ESR-related (CLE) peptides and the early nodulin gene, *ENOD40*. These promote nodulation. However, the soybean *euAP2* gene, *Nodule Number Control 1* (*NNC1*), has been shown to repress transcriptions of these genes by binding directly to their promoter, thereby inhibiting nodulation [116,117,118]. In addition, downregulation of *euAP2* genes either by overexpression of miR172 or RNAi leads to increased nodulation accompanied by upregulation of symbiotic leghemoglobin and non-symbiotic hemoglobin [119].

Similarly, downregulation of *euAP2* gene *RAP1* by miR172 facilitates tuberization in potato in a photoperiod-dependent manner [72]. Overexpression of miR172 hastens tuber formation under short days and stimulates tuber formation under long days. The overexpression of miR172 resulted in downregulation of *RAP1* in potato leaves. However, the downregulation of *RAP1* was not significant in stems and stolons of *35S:miR172* plants. While this suggests that miR172 does not downregulate *RAP1* in stems and stolons, it also hints at the possibility that miR172 may be acting on other potato *euAP2* genes that are yet to be investigated. Alternatively, miR172 may be promoting tuberization through a mechanism that is independent on its regulatory activities of *euAP2* genes.

## 5. Conclusions

*EuAP2* genes are broadly expressed in plants. In this review, we have summarized some of their reported roles in plant development besides flowering, for which they are well-known. It is possible that many other developmental effects of *euAP2* genes are not yet reported because of researchers’ focus on specific tissues. We, therefore, encourage a more holistic approach in the characterization of *euAP2* mutants. Such an approach will facilitate the understanding of their roles in plant development, and exploitation for domestication and biotechnological purposes.

## Figures and Tables

**Table 1:** Genome-wide content of AP2/ERF transcription factor superfamily in various plant species.

	Subfamilies					
Species	AP2	DREB/ERF	RAV	Soloist	Total	Reference(s)
*Actinidia deliciosa*	19	158	5	1	183	[22]
*Arabidopsis thaliana*	17; 18 *	121; 122 *	6	1	145; 147 *	[9,10] *
*Brachypodium distachyon*	23; 24 *	122; 112 *	4	0; 1 *	149; 141 *	[11,14] *
*Brassica rapa* ssp. pekinensis	29	248	14	1	291	[23]
*Bryum argenteum*	11	69	1	2	83	[24]
*Cucumis sativus*	20	103	4	4	131	[25]
*Capsicum annuum*	29	144	1	1	175	[26]
*Fagopyum tataricum*	15	116	3	0	134	[27]
*Glycine max*	26	120	2	0	148	[28]
*Hordeum vulgare*	19	95	6	1	121	[29]
*Jatropha curcas*	16	98	4	1	119	[30]
*Lotus corniculatus*	19	106	1	1	127	[31]
*Malus domestica*	51	195	6	7	259	[32]
*Medicago truncatula*	21	98	3	1	123	[33]
*Musa acuminata*	46	200	16	3	265	[34]
*Musa balbisiana*	49	243	22	4	318	[34]
*Oryza saiva* ssp. japonica	36	131	7	0	164	[9]
*Phaseolus vulgaris*	27	149	3	1	180	[35]
*Phyllostachys edulis*	28	80	7	1	116	[36]
*Populus trichocarpa*	26	168	5	1	200	[28]
*Prunus mume*	20	90	5	1	116	[37]
*Prunus persica*	21	105	5	1	129	[38]
*Ricinus communis*	19	90	4	1	114	[39]
*Salix arbutifolia*	22	145	4	1	173	[40]
*Setaria italica*	28	138	5	0	171	[41]
*Solanum lypersicon*	16	93	3	0	112	[42]
*Solanum tuberosum*	14	155	11	1	181	[43]
*Triticum aestivum*	9	104	3	1	117	[44]
*Vigna radiata*	16	55	2	1	71	[45]
*Vitis vinifera*	18; 20 *	109; 122 *	4; 6 *	1	132; 149 *	[46,47] *
*Zea mays*	22	107	3	1	107	[48]
*Ziziphus jujuba*	17	96	5	1	119	[49]
*Zoysia japonica*	10	131	6	0	147	[50]

* indicates source of second figure when two figures are presented.
